# Ethical tensions in the informed consent process for randomized clinical trials in emergency obstetric and newborn care in low and middle-income countries

**DOI:** 10.1186/s12910-019-0363-0

**Published:** 2019-04-27

**Authors:** Dan K. Kaye, Gershom Chongwe, Nelson K. Sewankambo

**Affiliations:** 1 College of Health Sciences, Department of Obstetrics and Gynecology, Makerere University, P.O. Box 7072, Kampala, Uganda; 2 Johns Hopkins University, Berman Institute of Bioethics, 1809 Ashland Avenue, Baltimore, 21205 USA; 3 Department of Epidemiology and Biostatistics, School of Public Health, University of Zambia, P.O. Box 50110, Lusaka, Zambia; 4 College of Health Sciences, Department of Medicine, Makerere University, P.O. Box 7072, Kampala, Uganda

**Keywords:** Emergency care research, Informed consent, Emergency obstetric and newborn care, Vulnerability, Respect for persons, Justice, Ethical tensions

## Abstract

**Background:**

There is unanimous agreement regarding the need to ethically conduct research for improving therapy for patients admitted to hospital with acute conditions, including in emergency obstetric care. We present a conceptual analysis of ethical tensions inherent in the informed consent process for randomized clinical trials for emergency obstetric care and suggest ways in which these could be mitigated.

**Discussion:**

A valid consenting process, leading to an informed consent, is a cornerstone of this aspect necessary for preservation and maintenance of respect for autonomy and dignity. In emergency obstetric care research, obtaining informed consent can be problematic, leading to ethical tension between different moral considerations. Potential participants may be vulnerable due to severity of disease, powerlessness or impaired decisional capacity. Time for the consent process is limited, and some interventions have a narrow therapeutic window. These factors create ethical tension in allowing potentially beneficial research while avoiding potential harms and maintaining respect for dignity, human rights, justice and autonomy of the participants.

**Conclusion:**

Informed consent in emergency obstetric care in low- and middle-income countries poses numerous ethical challenges. Allowing research on vulnerable populations while maintaining respect for participant dignity and autonomy, protecting participants from potential harms and promoting justice underlie the ethical tensions in the research in emergency obstetric and newborn care. Those involved in research conduct or oversight have a duty of fair inclusion, to avoid denying participants the right to participate and to any potential research benefits.

## Background

A valid informed consent process maintains and preserves respect for participant autonomy and dignity [1, 2] and protects research participants from potential risks and harms [2]. There are persistent debates on whether and when informed consent is necessary for some randomized clinical trials (RCTs) [3–7]. This stems from challenges of obtaining informed consent, partly from failure to comprehend disclosed information about the RCTs, yet this is necessary before participants can consider potential risks, benefits and alternatives to participation [8, 9]. This “understanding” [10] of research is necessary for *informed consent* but seldom happens [11–13]. Research should have social value and benefit to participants (or future patients), benefits of participation should surpass potential harms, and participant selection should be fair [9]. An ethical tension is a decision-making situation that necessitates choosing between two or more moral imperatives, neither of which is unambiguously satisfactory or preferable, and where obeying potentially results in transgressing another [14].

While high-quality obstetric delivery in health facilities has potential to reduce severe maternal and neonatal morbidity and mortality, its availability in many low- and middle-income countries (LMIC) settings, such as most of sub-Saharan Africa, is limited due to poor infrastructure, limited skilled manpower and high disease burden [15–19]. The commonest barriers to providing timely care are institutional factors which lead to long delays (in providing care) and compromised birth outcomes [15–19]. Substantial within-country economic inequalities in access to basic and emergency care exist within LMIC settings, secondary to a combination several factors. These include [15–20] inadequate or inequitable access to emergency obstetric care, limited household income, lack of transport, limited information on healthcare services/providers, low women’s self-esteem, lack of birth preparation, negative cultural beliefs/practices, ignorance about required obstetric health services, high cost of services and poor referral practices.

Human resources-related challenges in sub-Saharan Africa (include shortage of qualified and skilled staff, increased staff work load, burn out), and systemic and institutional failures (lack of essential medications, equipment, supplies or medications; limited infrastructure like operation theaters and high dependency units, and poor data collection and monitoring systems) [21–24] compound the problem of poor-quality healthcare. There is a high burden of acute illness in settings and contexts where acute care systems (that could significantly lower the morbidity and mortality) and integrated approaches (triage, resuscitation, stabilization and referral) are lacking to manage urgent and emergent conditions [24]. Research in emergency obstetric and newborn care in this context faces similar practical and ethical challenges. This paper presents potential ethical tensions inherent in the informed consent process for RCTs in emergency obstetric care and suggestions on how they can be mitigated.

## Main text

### Cognition and decisional capacity to consent for research in emergency obstetric care

A central concept of human research ethics is the value of respect for persons, which is most manifest in the informed consent [1, 2]. Respect for persons involves promoting and enabling individual participants’ freedom to make choices about participation, that is “… *capacity to advance and put one’s principles and values into practice”* [2], without constraints or undue influence [2]. Autonomy consists of two aspects [2]: a *volitional component*, whereby the decision is voluntary (not made by compulsion, threats or coercion), and a *cognitive component* (which requires that the individual has both the capacity and knowledge to make a decision about their intention). The fundamental premise that supports this requirement is that participation is not obligatory, and therefore inclusion in research ought to be the result of personal choice, according to participant preferences and values [1, 2, 10].

Researchers and research ethics committee members should understand the appropriate application of the principles (respect for autonomy, beneficence, non-maleficence and justice) in emergency obstetric and newborn care research in LMICs for several reasons. The first consideration is the emergency context. Clinical research in critically ill patients presents unique ethical considerations, from potential participants being a vulnerable population to participants presenting with complex physiologic problems that affect their cognition, comprehension (of disclosed information about research participation) or decisional capacity [25, 26]. Whereas they are at higher risk of harm, participants originate from a population that needs novel therapies that can reduce morbidity and mortality or alleviate suffering [25, 26]. This creates a tension between beneficence (benefitting from research outputs) and non-maleficence (potential risks and harms related to participation).

Besides inability to understand disclosed information and lacking decisional capacity to consent for themselves, critically-ill obstetric patients may fail to distinguish between aspects of clinical care and research, or may be vulnerable to exploitation or undue inducement [25, 26]. The latter two affect voluntariness of the consent process. This creates a conflict of competing values and moral implications where a (vulnerable) population with potential to ultimately benefit from research participation cannot benefit because they are excluded by (inability to voluntarily provide) consent for participation. Yet, it is the associated morbidities that might render them (vulnerable and) unable to provide informed consent (and therefor unable to participate in research) that are the main causes or predictors of mortality [26]. Potential participants are often in utmost need of innovative therapy, and many may be willing to assume some risk for potential benefit [26]. Due to the ethical tension between respect for autonomy, beneficence and non-maleficence, these patients require special safeguards to permit their participation in research.

Additionally, several potential barriers to inclusion of critically-ill participants in emergency obstetric care exist. Delirium, severe pain, hypovolemic shock or altered medical status, which impair their cognition, ability to understand disclosed information or decisional capacity, are part of the disease presentation. Besides, critically ill patients frequently undergo emergency treatment that affect their cognition (and therefore capacity to comprehend disclosed information) or ability to provide voluntary informed consent [27–30]. If informed consent is a necessary condition for research participation, such patients should be considered ineligible as potential research participants. Yet there is a compelling duty to involve patients (who may be unable to provide consent) in research in order to identify the best therapies for their illness [25, 26]. This creates tension between the need to respect participant autonomy and the compelling need (or necessity) to provide research benefits to needy populations, while avoiding participant harms.

### Mitigation of the ethical tensions in emergency obstetric and newborn care research

To mitigate the ethical tensions, ethical principles need to be specified, applied and balanced in specific contexts and situations. One key ethical tension is prohibiting potentially valuable research because informed consent is not possible (or is incomplete) versus enrolling individuals without informed consent. There are guidelines on when informed consent requirements may be waived during emergency research [26, 31]. Permitting certain emergency RCTs might provide individuals with life-threatening illnesses access to potentially life-saving therapies, advance knowledge through generation of data about effectiveness and safety, improve therapies used in emergency situations for which there are poor clinical outcomes [26, 31]. Such RCTs involves vulnerable population of participants, with potentially impaired capacity to provide consent, in a setting where the emergency circumstances require prompt action with limited time or opportunity to locate and obtain consent from each subject’s legally authorized representative [26, 31].

Surrogates may be used to provide *substituted judgment*, that is, to make decisions based on the known or perceived patients’ convictions rather than their own [25]. Surrogate consent for participation might be permissible and justifiable if there is inconsequential risk to the patient secondary to participation [25]. There are, however, debates about who can provide informed consent for a critically ill patient that is unable to consent for research. Could it be a spouse or other family member where the potential participants have no advance instructions regarding research participation? Family members have a lot of say in peoples’ lives (especially during pregnancy, childbirth or newborn care) and often women find it difficult to make decisions about their own lives [32, 33], more so in the more complicated situations like medical/obstetric emergencies [32, 33].

Women often refer to the husband, in-laws or parents for decision-making about healthcare decisions [33]. So spouses, if there are no advance instructions, may be a logical choice of who should provide substituted judgement for research participation. However, prospective research participants (or their surrogates) face the daunting challenge of distinguishing interventions for the emergency [26, 31] (for instance, resuscitation and stabilization of the patient as part of routine emergency care) from the research aspects of these interventions. Additionally, the emergency care setting is intensely emotional, and creates a state of “psychological dependency” [25]. Vulnerability may arise if excessive dependency leads to incapacitation for deliberation about voluntary decision to participate [25]. Often, critically ill patients and their families develop close relationships with their physicians [25]. Even brief information provided could inappropriately burden patients, relatives or parents when they are highly stressed, and thus may need to consult their physicians first for opinion [25]. Surrogates (dealing with the emotional, psychological and logistic impact of hospitalization of a loved one) may not fully comprehend the disclosed information about the RCTs to provide consent in the best interests of the patient [25, 34].

For researchers, the balance between protection and inclusion of potential research participants causes ethical tension. For researchers, an invalid “informed” consent process presents ethical tension between the principles of respect of persons (respect for autonomous decision-making) and beneficence (generating data to address critical research questions on improving healthcare in an ethical manner). Another inherent tension exists between concerns from potential participants (who may decline participation) and maximizing numbers enrolled [33, 34]. Providing detailed information may deter comprehension and potential participation, and limited time between consent and participation may not allow detailed discussion with prospective participants or their surrogates [35, 36]. This scenario may create ethical tension for clinician-investigators, secondary to conflict of interest, in addition to the psychological dependency [25]. Suggestions by these to potential research participants to enroll in research may “blur boundaries” between usual care and research [25, 27], thereby putting into question the validity of the informed consent process regarding its voluntary character. This commonly occurs in relation to powerlessness and the power imbalance between physicians and their patients, and where consent is negotiated through a relationship in which the potential participant is dependent on the clinician-investigator.

There are possible solutions to the above problem: a) having potential participants discuss their decisions with someone who is potentially able to support them in reaching a decision and, b) arranging for someone other than the investigators to negotiate the consent. While a dependent relationship by itself ought not invalidate decision to participate [2], there should be strategies to address the potential effects of such relationships on validity of the consent process [2, 9]. Positive relationships between potential research participants (or their family members) and researchers may enhance individual’s freedom to make independent choices [2]. However, in other situations, the power imbalance between investigators and potential participants may compromise validity of the consent process by either causing undue influence or exploitation of trust [2]. Having a different person other than the clinician-investigator conducting the recruitment process may reduce the power imbalance, reduce therapeutic misconceptions, and strengthen the trust in the patient-provider relationship, much as it may not remove the undue influence [2].

The Declaration of Helsinki [26] addresses the dilemma of research without consent by allowing a waiver or modification of informed consent in some RCTs. For RCTs among individuals that are incapable of giving informed consent, the Declaration of Helsinki [26] gives guidance where the need or procedures for informed consent may be modified as manifested in some research in emergency situations [27–32]. The Declaration states that if no surrogate or patient representative is available and the research cannot be delayed, the study may proceed without participant consent under certain conditions: a) the specific reasons for involving patients as RCT participants is a disorder that renders them unable to give informed consent explicit in the research protocol; and b) the study protocol is approved by a research ethics committee. The conditions for which RCTs may be necessary in emergency obstetric care (such as eclampsia, antepartum hemorrhage and obstructed labor) exist only in pregnancy, and more often as emergencies. Also, the complications may cause severe morbidity, which, as well as ongoing treatment (such as for pain), may be the reason for impaired cognition or decision capacity.

There are additional suggestions on how to ethically and justifiably conduct research without prior consent. The participants are vulnerable to the illness, the risks of research participation, and the risk of potentially being denied (including all future patients) beneficial therapy when no effective treatment exists [35–37]. The permissibility of such research requires that the research has high social value, is conducted with utmost rigor, that potential risks of participation are minimized and participants’ well-being and welfare are promoted to gain a favorable benefit-risk ratio, and all possible protections (including ethics committee oversight) are maximized [9, 26, 32]. Also, different randomization protocols for which informed consent may not always be necessary can be considered, especially for pragmatic RCTS [38]. Here, treatment options may be randomized according to time or to research site, in a transparent manner, and individual patients (in some situations) may even be randomized to different treatment options consecutively, where they serve as their own control [38]. There might be need for ‘de-juridification’ of the information process between a clinician/researcher and a patient [38]. Here clinicians may recruit patients ass research participants without consent as long as the RCT is testing proven interventions, patients are reasonably informed and accept that clinicians can propose treatment strategies according to their judgment, and risks are minimized [38].

Another situation where waiver of informed consent may be applied is comparative effectiveness trials of known medications or procedures [39]. Informed consent may not always be needed in therapeutic RCTs where benefit is anticipated for every individual participant, for instance, where participants are randomized to one of several already approved therapies, especially when they are of similar nature and have similar guideline recommendations [39]. This may be necessary where informed consent is not feasible or possible, and provided procedures are instituted to minimize harm and maximize benefit, participants are carefully monitored, and the RCT is approved prior and monitored by ethics committees [39].

Exceptions to informed consent may be permissible in emergency obstetric care research, especially pragmatic RCTs [37–39]. Additional guidelines can enable researchers to recruit participants (even where it is not feasible to obtain prospective or proxy consent for emergency research) by considering whether research (without initial consent) could be justifiable. This depends on whether the values that are protected by the informed consent (respect for autonomy and dignity) can be secured by or replaced by other values. In the consent-substituted model [37], replacement values include *responsiveness* (the intervention ought to be responsive to an urgent medical need), *favorable risk-benefit ratio*, absence of *conflicting preferences* (no compelling reason to believe that participation in the research conflicts with enrolled patients’ values or interests), minimal *net risks* (non-beneficial procedures cumulatively pose no greater than minimal risk), and *prompt consent* (consent for ongoing and additional interventions) is obtained as soon as it is possible or feasible.

Exceptions to informed consent may be permissible in emergency obstetric care research, especially pragmatic RCTs [37–39], and where it is possible to conduct community consultations [40]. This approach may be more practical as it provides critical guidance for the conduct of research in learning health systems (where the generation of new knowledge, whilst important, is embedded in ongoing medical practice). Community consultations could be used to enable investigators or institutional review boards to obtain community input regarding planned emergency research, facilitate community understanding, promote trust, and ensure justice and eventual protection of research participants [40]. The challenge is that this process requires active participation by community members, does not seek approval, consent or consensus, and is faces lack of clarity of are the appropriate community representatives or which approaches are effective for engaging them [40]. While ethically acceptable, using surrogates or community consultations becomes practically challenging for several reasons: First, few people discuss in advance their preferences and values regarding participating in research, so surrogates can only guess the best patient interests [41–43]. Secondly, alternatives to participation may be limited [41] as the medical product or procedure may only be available to clinical trial participants [42, 43].

Lastly, investigators using pragmatic trials may employ the integrated consent model of informed consent. This may be used for pragmatic trials comparing commonly used treatments which are already in routine practice, where the researchers normally would require only verbal consent [44]. The approach integrates clinical and research consent within the same clinical encounter, whereby the attending physician will inform the patient about the treatment’s rationale, alternatives, use of randomization, potential harms and benefits of the therapies under comparison [44]. The patient can then opt out through oral or written consent.

### Balancing the interests of the mother and fetus

There is ethical tension in balancing the interests of the mother and fetus (or eventually the newborn) in RCTs in pregnancy or perinatal research. Research may be directed towards a condition of the fetus/newborn and with prospects of direct benefit to the fetus/newborn (and none to the mother), or research may be directed towards a problem of the pregnant woman with anticipation of direct benefit to the woman alone (or to both mother and fetus/newborn) [45]. In the former, there is concern over how risks to the fetus should be balanced against anticipated benefits [45]. In the latter, there is concern on whether potential risks to the mother are reasonable or can be minimized [45]. In either case, the risks to the fetus should be reasonable in relation to anticipated benefits [45], yet potential risks to the mother can be reduced by increasing potential risks to the fetus (or newborn) and vice versa [45]. This underscores a need to ensure that any risk should be the least possible for achieving the research objectives [45], and if there are alternative ways to reasonably and satisfactorily achieve the research objectives, the least risky alternative to both mother and fetus/newborn should be selected [45]. There is need, in addition, to ensure that the context of emergency obstetric care does not significantly add to the potential research-related risks and harms.

Specifically for RCTs in emergency obstetric care, the CIOMS guidelines [8] offer additional guidance: a) Research could be conducted on pregnant women if there are potential direct benefits to the pregnant woman and the risks to the fetus and pregnant woman are minimal; b) Clinical trial-related risks (which may sometimes compound the background risks and assessments of foreseeable potential risks) need, where possible, to be communicated to prospective participants [8]; c) Low literacy levels and failure to understand concepts (such as blinding, randomization and equipoise) may not add higher risks than the baseline existent risk. Therefore, inability to comprehend these concepts does not necessarily render the informed consent unacceptable or the RCT unethical [8], particularly where there is compelling need to conduct the RCT. Thus, the Declaration of Helsinki [26] and the CIOMS guidelines [8] address the issue of justice (in excluding potential participants where individual or proxy consent is not possible, denying them the right to participate and to any potential benefits), especially in contexts with weak emergency healthcare systems. Also, in some situations, this opportunity to participate may be the only way to ensure that treatment for emergency obstetric care complications is available [8]. Besides, it may only be through opportunity for research participation that the communities obtain the basic infrastructure for healthcare [8] (such as neonatal intensive care units, incubators and resuscitation equipment).

Fair inclusion may also be used as justification for including pregnant women in research [45]. Fair inclusion implies that pregnant women who are eligible should not be excluded solely for being pregnant, (and arguably for having pregnancy complications) and that interests of pregnant women are prioritized [45]. This suggests that RCTs in emergency obstetric care may be ethically permissible as long as precautions are taken to ensure a favorable benefit-risk ratio and scientific rigor [45]. The RCTs are permissible as effects of interventions in pregnancy may differ from effects in other sub-populations (such as non-pregnancy state) [45].

For RCTs in obstetric care, where complications have insidious onset or slow progression, a tiered-layered or staged process (similar to the multi-level multi-stage model suggested for informed consent for genomic research [46] and neonatal screening [47]) may be employed. One level or stage might be providing information and clarifying any issues to the patient, spouse or other relatives of the potential RCT participant. This stage could occur at any stage during pregnancy or childbirth, before the severe complications ensue. If the prospective participant (or their surrogates and relatives) register no objection to recruitment in RCTs, the participant or their surrogates are given more specific research-related information, and specific consent is sought. The process of engagement could go on until the potential participant is enrolled into the RCT. If the potential participant or their relatives/surrogates object at the preliminary or latter stages, the patient should not be considered further for inclusion in RCT, (unless they approach investigators on their own). The multistage consent process [45], while potentially able to provide opportunity to overcome ethical barriers to research without initial consent, raises concerns on how the delays inherent in this process can be reduced for RCTs in emergencies. It however has some merit. For instance, sensitizations of pregnant women (as potential RCT participants in case obstetric emergencies) could be initiated earlier, for instance in early labor for intrapartum complications. Such sensitization could occur during antenatal care (for problems known to recur such as pre-eclampsia or postpartum hemorrhage) or in early labor for known complications of late stages of labor.

### Addressing the research context as a matter of human rights

Human rights violations play an important role as determinants of, or structural barriers to, health, and research on human rights ought to lead to development of rights-based interventions and the promotion of human rights [47]. The aspects of health as a human right include the indivisibility of civil, political rights and socio-economic rights, recognition of active agency by populations that are vulnerable to human rights violations; and the strong normative role of human rights in establishing accountability for protections and freedoms [47]. If emergency obstetric care primarily ought to triage, resuscitate and stabilize such patients [24, 47], absence of the necessary requirements to achieve (or failure to promote research directed at these outcomes) [48] in LMIC emergency healthcare contexts constitutes a human rights problem. From human rights considerations, clinician-investigators have a moral obligation to provide opportunity for individuals seeking emergency care to participate in potentially beneficial research [47]. Researchers ought to highlight and the obstetric problems that individuals present with, and address them to the best of their ability, despite the limitations of the healthcare system [24, 48], and remind the state as duty bearer of the duty to protect the right to health. RCTs are an addition to strategies for progressive realization of this obligation, by providing necessary data (such as for essential medicines). This creates an ethical tension between ensuring access to potentially beneficial research and creating additional burdens for individuals in a care-research environment [24] that lacks basic necessities. Yet, where opportunities for RCT participation are available, delay to access care due to long consent procedures (in emergency situations) may result into avoidable morbidity (and probably mortality) or delays in accessing potentially beneficial treatment [27].

## Conclusion

The informed consent process for RCTs in emergency obstetric care in LMICs is beset with ethical tensions related to promoting respect of persons, promoting beneficence and avoidance of harms. The complex ethical issues show that existing ethical guidelines could be interpreted in multiple ways, and that competing principles ought to be balanced against each other. In the conceptual analysis, the ethical tensions that arise in the informed consent process in emergency obstetric care are important and need to be recognized. There is a compelling need to consider different ways in which pregnant women could be recruited in RCTs that have potential to benefit them or similar populations**.** Investigators seeking to conduct RCTs in such contexts need to conduct an ethical analysis of the appropriate alternatives so as to promote autonomy, justice, beneficence and human rights of the potential RCT participants.

## Abbreviations

CIOMS: Council of the International Organization of Medical Sciences
LMICs: Low-and Middle-income countries
RCTs: Randomized Clinical Trials
